# Nanoscale rotary apparatus formed from tight-fitting 3D DNA components

**DOI:** 10.1126/sciadv.1501209

**Published:** 2016-02-19

**Authors:** Philip Ketterer, Elena M. Willner, Hendrik Dietz

**Affiliations:** Physik Department and Walter Schottky Institute, Technische Universität München, Am Coulombwall 4a, 85748 Garching bei München, Germany.

**Keywords:** Biophysics, rotary motors, self-assembly, single-molecule, DNA origami

## Abstract

We report a nanoscale rotary mechanism that reproduces some of the dynamic properties of biological rotary motors in the absence of an energy source, such as random walks on a circle with dwells at docking sites. Our mechanism is built modularly from tight-fitting components that were self-assembled using multilayer DNA origami. The apparatus has greater structural complexity than previous mechanically interlocked objects and features a well-defined angular degree of freedom without restricting the range of rotation. We studied the dynamics of our mechanism using single-particle experiments analogous to those performed previously with actin-labeled adenosine triphosphate synthases. In our mechanism, rotor mobility, the number of docking sites, and the dwell times at these sites may be controlled through rational design. Our prototype thus realizes a working platform toward creating synthetic nanoscale rotary motors. Our methods will support creating other complex nanoscale mechanisms based on tightly fitting, sterically constrained, but mobile, DNA components.

## Teaser

A nanoscale rotary mechanism reproduces some of the dynamic properties of biological rotary motors.

## INTRODUCTION

Biological macromolecular machines can accomplish complex tasks such as transport and catalysis. The bacterial flagellar motor (*1*–*3*) and the F_1_F_0_–adenosine triphosphate (ATP) synthase (*4*–*6*) are two rotary machines that fascinate in particular through their intricate structure and impressive performance. It appears hardly imaginable today that mankind could ever devise synthetic nanomachines that achieve similar functionalities.

**Fig. 1:**
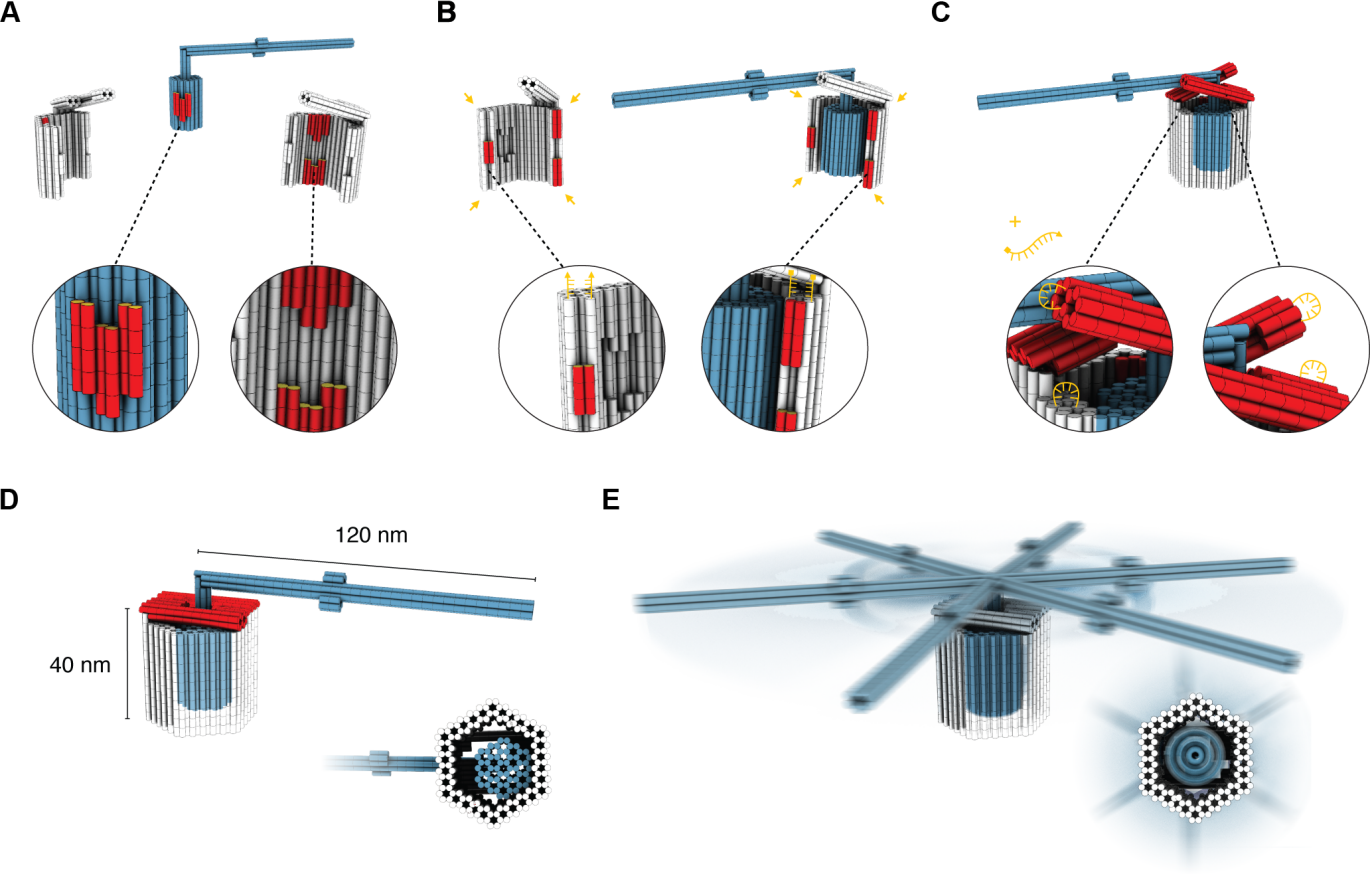
Design of a DNA-based rotary apparatus. (**A**) Schematic parts list. Blue, rotor unit; gray, clamp units; red, shape-complementary sockets on rotor and clamps. The rotor body consists of 54 parallel 94-bp-long double-helical DNA domains (see fig. S1 for design details) packed on a honeycomb-type lattice. The clamps consist of 62 parallel 115-bp-long double-helical DNA domains, also packed on a honeycomb-type lattice (see fig. S2 for design details). Cylinder elements indicate double-helical DNA domains with one turn length. (**B**) Red, shape-complementary sockets for docking the remaining clamp to the rotor-clamp dimer; yellow, complementary single-stranded DNA sticky ends (15 bases each) for reinforcing the bond between the clamp elements. (**C**) Bracket (red) closure through hybridization of auxiliary oligonucleotides that bind to single-stranded DNA loops in the clamp elements to connect the brackets to the respective opposite clamp element. (**D**) Fully assembled trimeric rotor apparatus with closed brackets and a docked rotor. (**E**) Fully assembled trimer with an undocked, mobile rotor.

A combination of recent advances with sequence-programmable self-assembly of DNA (*7*–*10*), however, enabled us to take a step toward creating such machines. We fabricated various prototypes of passive nanoscale rotary mechanisms with unprecedented complexity. Molecular self-assembly with DNA was previously used to build nanoscale mechanisms (*10*–*14*), but remaining strand linkages or other design features limited the range of domain movement within those mechanisms. Our apparatus is designed to tightly constrain the motion of a rotor to one rotational degree of freedom but without restricting the angular range of rotation. To this end, we constructed the rotary apparatus from three different multilayer DNA origami (*9*, *15*, *16*) components (Fig. 1): a rotor unit and two clamp elements that form an axle bearing. The body of the rotor unit is ~32 nm long and has a hexagonal cross section. The diameter of the envelope cylinder around the rotor body is approximately 22 nm. The rotor unit also features a crank lever–like axial protrusion. The clamp elements are ~38 nm long and have a cross section resembling a bisected hexagon. The clamp units each feature a bracket-like protrusion.

For assembly (see movie S1), we first dock the rotor onto one clamp unit (Fig. 1A) via a shape-complementary “socket” (*10*). To close the axle bearing, we click the second clamp element into another socket on the clamp that already hosts the rotor (Fig. 1B). Hybridization of sequence-complementary DNA single strands distributed across the clamp elements’ mutual binding interfaces strengthens the bond between the two clamp units. We then tighten the brackets on the crank lever–facing opening of the cavity through the addition of auxiliary DNA single strands (Fig. 1C). The brackets rigidify the bearing and irreversibly trap the rotor in the cavity.

Our apparatus realizes a mechanically interlocked architecture (*17*, *18*). The bearing cavity leaves a 22-nm-wide rotation tolerance between its walls and a centrally placed cylindrical envelope of the rotor unit. Off-axis tilt movements of the rotor relative to the bearing are therefore suppressed to ~7° for flat-to-vertex and to ~14° for flat-to-flat side rotor orientations within the hexagonal cavity. As we will show, depending on the solution conditions and the bearing variant used in the apparatus, either the rotor can dwell in docking sites (Fig. 1D) or it may perform rotary random walks around the central axis of the bearing (Fig. 1E; see also movie S2).

## RESULTS

**Fig. 2:**
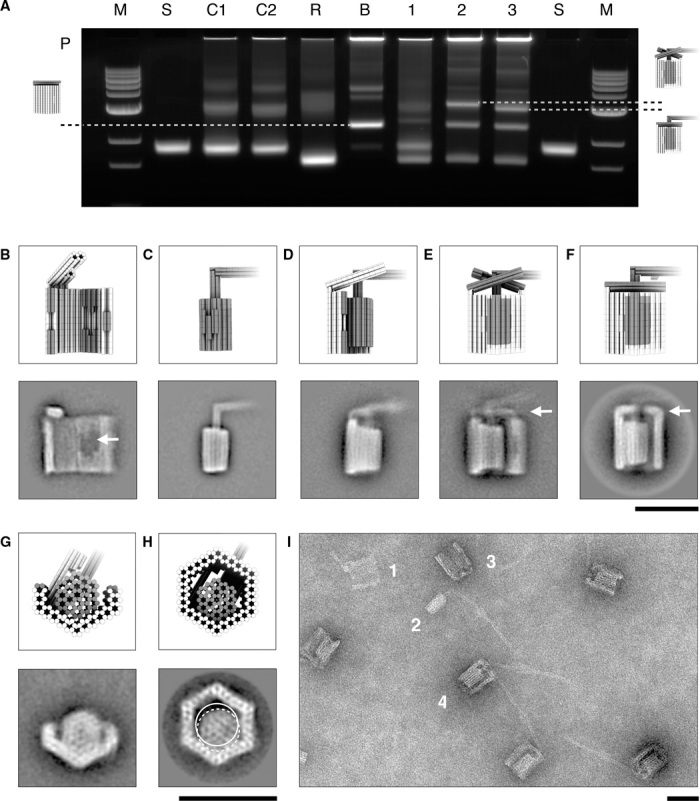
Assembly of the rotary apparatus. (**A**) Laser-scanned image of a 2% agarose gel with 11 mM MgCl_2_ run on an iced water bath on which the following samples were electrophoresed: M, 1-kb ladder; S, scaffold DNA; C1, clamp element 1; C2, clamp element 2; R, rotor unit; B, bearing dimer; P, pocket; 1, rotor and clamp dimerization; 2, bearing closure reaction in which the second clamp element was added to the mixture in 1; and 3, bracket closure reaction in which auxiliary oligonucleotides were added to the mixture in 2. (**B** to **H**) Schematic representation and average negative-stain transmission electron microscopy (TEM) micrographs: clamp element (B), rotor unit (C), rotor-clamp dimer (D), trimer consisting of rotor and both clamp elements with loose brackets (E), and trimer with fixed brackets (F). White arrows indicate the shape-complementary docking site in (B) and positions of brackets in (E) and (F), respectively. (G and H) Axial views of the rotor-clamp dimer and the trimer with fixed brackets, respectively. White circular lines indicate the circumscribed circle of the hexagonal cross section of the rotor body in the docked (dashed line) and undocked position (solid line). (**I**) Exemplary negative-stain TEM micrograph acquired from sample 3 in (A): 1, single clamp element; 2, single rotor unit; 3, empty bearing dimer with fixed brackets; and 4, fully assembled trimer with fixed brackets. Scale bars, 50 nm.

We used electrophoretic mobility analysis (EMA) in agarose gels to evaluate the assembly products on the ensemble level. The clamp element dimerization reaction proceeds with high yield independent of the presence of the rotor, as evidenced by a bright clamp dimer band (Fig. 2A, lane B). Dimerization of the rotor with one clamp element was performed in the presence of 30 mM MgCl_2_. EMA of the products in the presence of 22 mM MgCl_2_ reveals a new band with mobility in between those produced by the clamp dimers and monomers (fig. S3, lane 1). We attribute this band to rotor-clamp dimer complexes. During EMA of the dimerization products in the presence of 11 mM MgCl_2_, we did not observe such dimer band, indicating that the complex dissociated during electrophoresis (Fig. 2A, lane 1). These data show that cation concentration modulation is a viable strategy for controlling rotor release after assembly. Adding the second clamp unit to the rotor-clamp dimer at 30 mM MgCl_2_ yields a new, lower mobility band (Fig. 2A, lane 2), which we identify with the trimer complex. Hybridization of the auxiliary DNA single strands for fixing the brackets enhances the mobility of this species (Fig. 2A, lane 3), which supports the success of the bracket closure reaction.

We used direct imaging with negative-staining TEM to study the shape of assembly products on the single-particle level. The appearances in TEM images of the monomeric components for our rotary device (Fig. 2, B and C), of the rotor-clamp dimers (step 1; Fig. 2D), of the rotor-bearing trimers with mobile brackets (step 2; Fig. 2E), and of the final rotary device with fixed brackets and rotor docked in an off-center position (step 3; Fig. 2F) that we acquired are all in agreement with our expectations. With fixed brackets, the bearing is more resilient against stain-induced flattening in TEM (see also fig. S4). TEM projections obtained along the helical axis of rotor-clamp dimer particles verify the correct placement of the rotor into the docking site (Fig. 2G). TEM projections taken from the final trimer along the helical axis (Fig. 2H) confirm that the diameter of the bearing cavity is sufficiently large to permit rotation of the rotor. Field-of-view TEM data of unpurified reaction mixtures obtained after completion of the assembly protocol (as electrophoresed in lane 3 of Fig. 2A) reveal trimer particles with the crank lever protruding in the designed orientation through the bracket-reinforced opening in the bearing. The images also show the designed 90° kink in the lever arm (Fig. 2I).

Previously, Noji *et al*. (*19*) and Yasuda *et al*. (*20*) revealed the rotary mechanism of ATP synthase through a series of challenging single-particle experiments. In these experiments, fluorescently labeled micrometer-long actin filaments were rigidly attached to the shafts of individual F_1_–ATP synthase molecules. The labeled filaments produced a pointer-shaped fluorescent signal that could be observed with diffraction-limited optical microscopy and that directly reported on the angular orientation of the F_1_–ATP synthase rotor. Inspired by these experiments, we used analogous single-particle assays to explore the dynamics of our rotary apparatus, to study rotor and bearing design variants, and to test the influence of solution conditions on rotor dynamics.

**Fig. 3:**
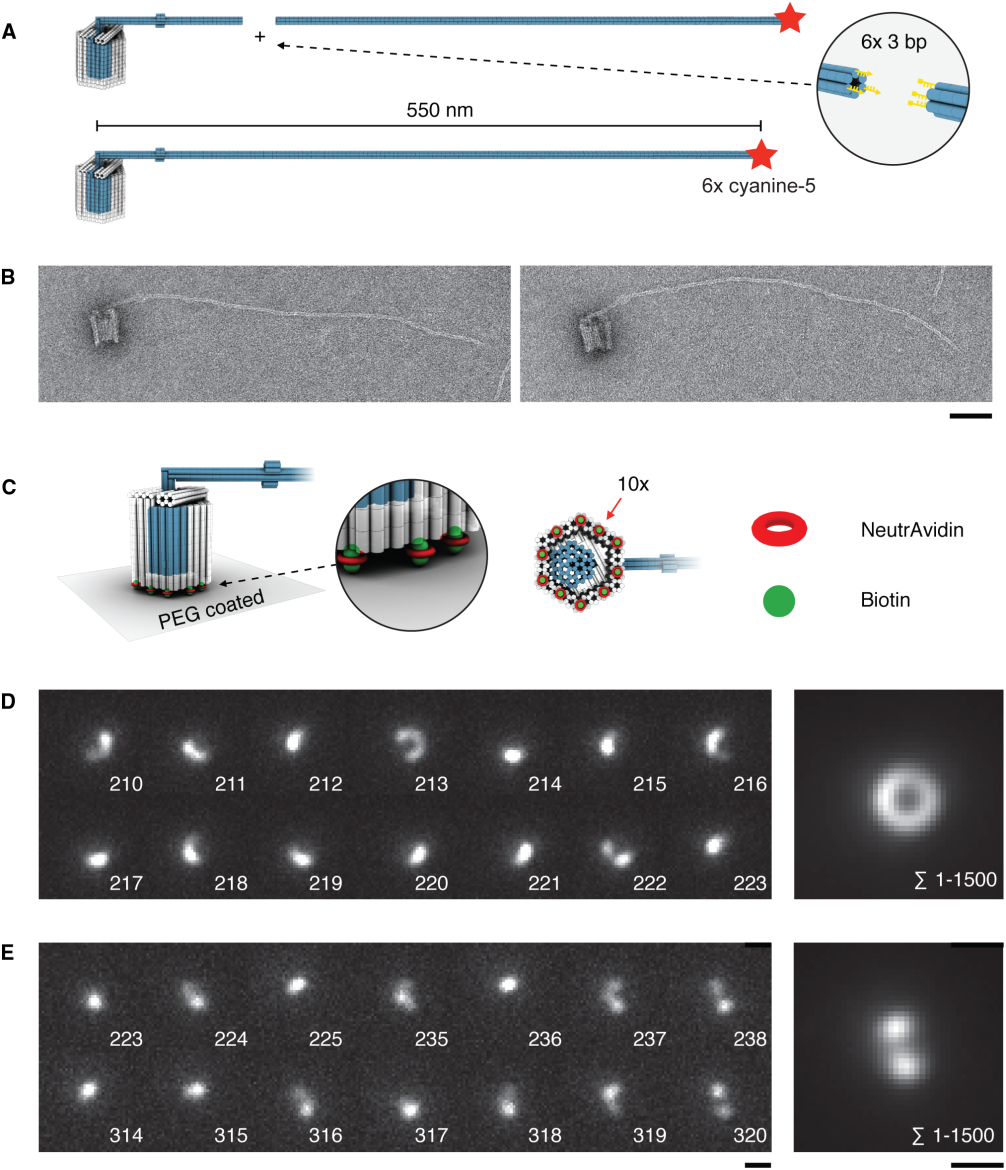
Experimental proof of rotary movement. (**A**) Schematic illustration of the crank lever extension using a 430-nm-long six-helix bundle unit (see fig. S8 for design details). (**B**) Negative-stain TEM micrographs of a fully assembled tetramer. Scale bar, 50 nm. (**C**) Schematic representation of surface attachment of the apparatus for fluorescence microscopy. Coverslips are coated with unmodified and biotin-modified PEG. Ten biotin-modified oligonucleotides are incorporated into the bearing at the indicated positions to form multiple biotin-NeutrAvidin-biotin linkages per apparatus with the surface. (**D** and **E**) Left: Exemplary single frames of two single-particle fluorescence microscopy recordings. The measurements were acquired in total internal reflection (TIRF) mode in the presence of 5 mM MgCl_2_. Right: Sum over all 1500 images taken in the respective single-particle recording. Exposure time is 50 ms. Scale bars, 1 μm (see figs. S9 and S10 for all frames).

For one set of experiments aimed at demonstrating the rotary mechanism, we extended the crank lever of the rotor unit with a 430-nm subpersistence-length DNA origami filament (Fig. 3A and fig. S5). By design, the circle circumscribed by the extended crank lever has a diameter of ~1 μm (see movie S3). TEM images confirm the correct assembly into the desired shape (Fig. 3B and fig. S6). To enable readout of the rotor orientation in single-particle fluorescence microscopy, we labeled the extended crank lever at its tip with six cyanine-5 reporter dyes (Fig. 3A). To attach the apparatus to microscopy surfaces in a rotationally constrained fashion, we placed 10 biotin moieties across the lower helical bearing interface (Fig. 3C) so that multiple bonds with a NeutrAvidin-coated surface may be formed. We purified the biotinylated and fluorescently labeled 20-MD heterotetrameric apparatus from agarose gels, applied the extract to NeutrAvidin-coated and polyethylene glycol (PEG)–passivated microscopy glass slides, and studied the samples in a home-built single-particle fluorescence microscope (Supplementary Methods).

In the video data thus obtained from single rotary apparatus particles, we observed frequently arc-like optical signatures in individual frames (Fig. 3, D and E, left). One group of single-particle recordings shows arc-like optical signatures in the majority of frames per video (movie S4). Summing up all frames in such videos produces a donut-like optical signature with a diameter of ~1 μm (Fig. 3D, right). We observed particles producing such donut sum signatures independent of whether we used evanescent or epi-illumination excitation and in parallel to particles that produced filled-circle–like sum recordings (fig. S7). Another group of single-particle recordings shows arc-like signatures in the minority of frames per video (movie S5). Summing up all frames in these recordings produces a more double-spot–like optical signature with a center-to-center spot distance of ~1 μm (Fig. 3E, right).

Given the close correspondence with the designed dimensions of our apparatus, we interpret the arc-like signatures as reflecting rotary movements in which the crank lever tip sweeped a circular arc parallel to the surface during acquisition of a frame. For example, the exemplary sequence of frames in Fig. 3D initially suggests cumulative ~600° clockwise rotation during frames 210 to 215, followed by rotations that change direction from frame to frame. We attribute dwells in between the rotary movements (Fig. 3E, left) to transient docking of the rotor into the sockets located at opposing faces within the bearing. Switching from one socket to the other requires a 180° rotation, which leads to 1-μm displacement of the crank lever tip in agreement with our data (Fig. 3E, right). Together, the data thus provide direct proof of the rotary mechanism of our apparatus.

Previous experiments with single filament–labeled ATP synthases revealed a significant degree of surface-induced heterogeneity: About 80% of the particles were immobile, about 20% of particles showed some movement, and a yet smaller fraction (1 of 70 particles) showed active rotation (*19*). We also observed heterogeneity. About 80% of single-particle recordings that we made with the extended crank lever did not show any dynamics at all, which we attribute to particles where the long lever arm got stuck unspecifically on the surface. The filled-circle–like recordings mentioned above within the mobile class of particles suggest movement of the lever tip on the surface of a half sphere, instead of on a circle. This may be explained by particles that have formed only one NeutrAvidin-biotin bond (instead of multiple bonds) with the surface. Finally, the heterogeneity with respect to rotor docking in our recordings (Fig. 3, D versus E) may be attributed to deformations of the bearing upon binding to unfavorably distributed NeutrAvidin molecules. Shape alterations may induce a geometric deactivation of the docking sites in the bearing, as previously seen with other shape-complementary DNA objects (*10*).

**Fig. 4:**
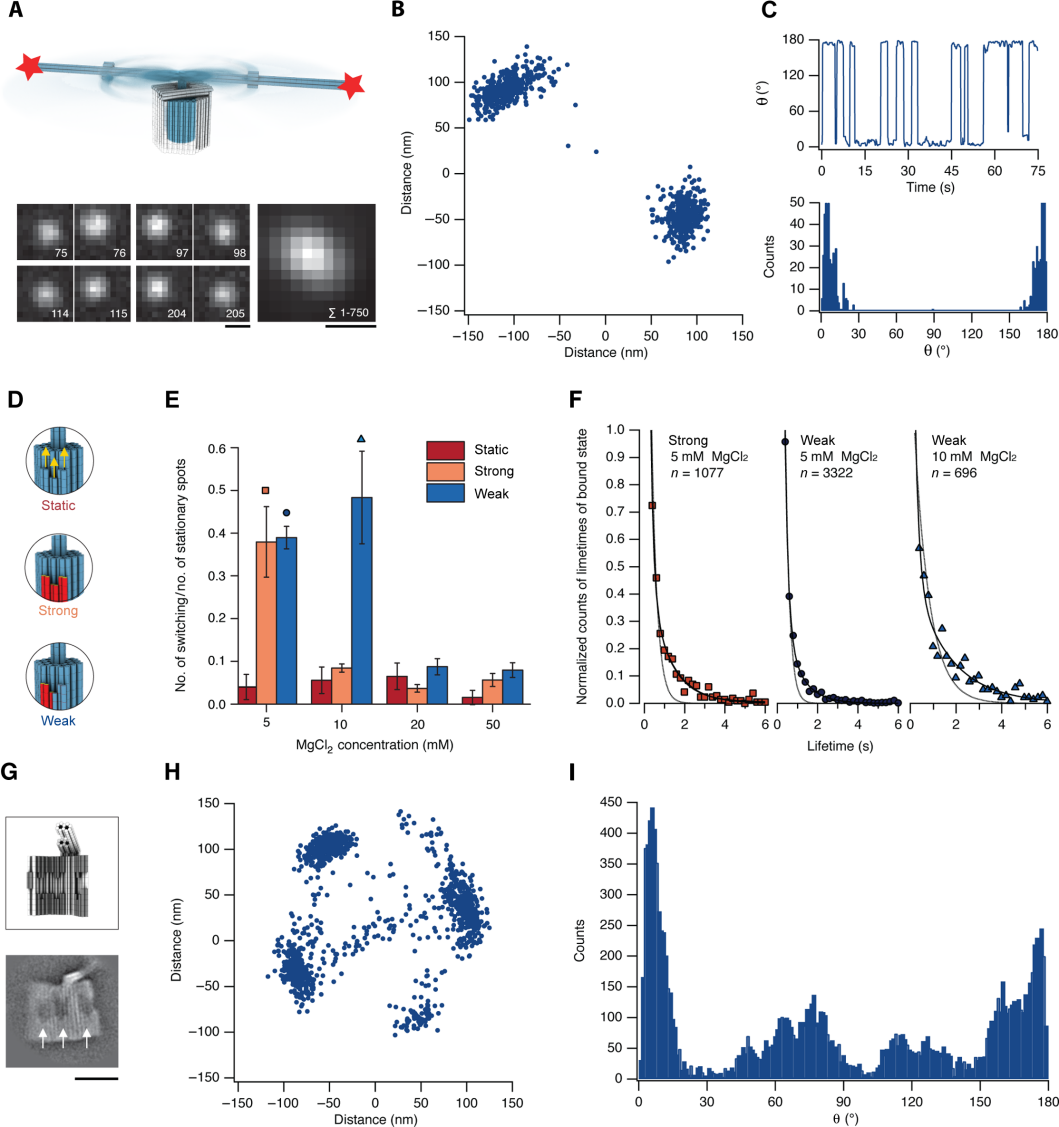
Rotor docking dynamics. (**A**) Top: Schematic view of the rotary apparatus with a 120-nm-long crank lever, showing overlay of two possible docked states and intermediate rotary states. Stars, six cyanine-5 dyes at the tip of crank lever. Six additional cyanine-3 dyes were placed as an immobile position reference at the crank lever kink (not indicated in the scheme). Bottom left: Exemplary single frames of a single-particle fluorescence microscopy movie, acquired in the cyanine-5 emission band in TIRF in the presence of 5 mM MgCl_2_, using alternating laser excitation (ALEX) (*29*). Exposure time per channel was 50 ms. ALEX was used to help discriminating mobile rotors. The frame sequence illustrates consecutive switching events. Bottom right: Sum over all 750 images in the recording. Scale bars, 0.5 μm (see fig. S12 for all frames). (**B**) Scatterplot of rotor tip coordinates obtained through centroid tracking of the recording shown in (A). (**C**) Time evolution (top) and histogram (bottom) of the angle θ calculated from data in (A). θ is defined as the angle between the origin in the scatterplot and the fitted coordinates in (B) (see fig. S13 for further spots). (**D**) Schematic representation of three rotor versions with different affinities of docking sites. Static variant: Sticky ends (12 × 4 bases long) protruding from the rotor can hybridize to single-stranded scaffold loops present in the clamp elements. Strong variant: All oligonucleotides forming the rotor docking socket are incorporated to enable formation of up to 12 stacking bonds upon docking. Weak variant: Six of 12 oligonucleotides involved in the rotor docking socket were shortened such that only up to six stacking bonds may be formed upon docking (see figs. S1, S14, and S15 for design details). (**E**) Statistical ratio of switching versus nonswitching spots as observed in single-particle fluorescence microscopy for the three variants. Switching was defined as the occurrence of at least one back-and-forth switching event. The total number of spots analyzed after sorting out artifactual recordings (bleaching, drifting, etc.) is 9490. (**F**) Symbols: Histogram of docking dwells in the presence of 5 mM MgCl_2_ for the weak and strong version (30 and 25 particles analyzed) and for the strong version in the presence of 10 mM MgCl_2_ (17 particles analyzed). Solid lines, double-exponential fits; dashed lines, single-exponential fits. Mean durations of docking dwells: weak version 5 mM MgCl_2_, 0.6 s (SD, 1.8 s); strong version 5 mM MgCl_2_, 1.6 s (SD, 3.6 s); and weak version 10 mM MgCl_2_, 1.7 s (SD, 2.4 s). (**G**) Top: Schematics of a clamp variant with three sockets for rotor docking. Bottom: Average negative-stain TEM micrograph of the clamp variant. White arrows indicate docking sites. Scale bar, 50 nm (see fig. S16 for design details and fig. S17 for electrophoretic analysis). (**H**) Scatterplot of rotor tip coordinates obtained through centroid tracking of one exemplary single-particle recording, obtained with the six-docking-site bearing assembled from two clamp units as in (G). (**I**) Average angle histogram of θ calculated as in (C) from 11 individual single-particle recordings, where θ = 0 was placed at the cluster with highest occupancy.

For another set of experiments aimed at testing the docking dynamics of our apparatus, we used the trimeric rotor apparatus with the shorter crank lever (Fig. 1D), which led to an increased fraction of mobile particles. Arc-like signatures per frame can no longer be observed with the trimeric apparatus because of the subdiffraction length of the crank lever and its significantly enhanced rotational diffusion. However, docking site switching can be readily discriminated in single-particle recordings and evaluated as a function of time via centroid tracking (*21*) (Fig. 4, A to C).

The greater the strength of the designed docking interaction between the rotor and the bearing (Fig. 4D), the lower the cation concentration necessary for stabilizing the bond between them (fig. S11) and for quenching transition dynamics (Fig. 4E). When we used high-affinity hybridization-based bonds between rotor and bearing, we did not observe switching particles beyond a low, constant background ratio at any of the cation concentrations tested. We attribute the background to particles with defects in the docking sites. The average lifetimes of the docked states were on the scale of seconds (Fig. 4F). The average length of rotor dwells increased when docking sites with more designed stacking bonds in the bearing were used or when the cation concentration in solution was increased. We observed that rotor dwell times are distributed in a double-exponential fashion (Fig. 4F). This finding hints at sample heterogeneity, which may be attributed to chemical heterogeneity within the docking sites such as DNA oligonucleotide truncations or may be due to surface-induced deformations as explained above. When introducing six instead of two shape-complementary docking sites into the bearing (Fig. 4G), single-particle recordings displayed multistate switching (Fig. 4H) on up to six dwell positions spaced out on a circular ring (Fig. 4I) in approximately 60° angular increments.

## DISCUSSION

Together, our experiments demonstrate the successful construction of a passive, structurally well-defined, nanoscale rotary mechanism. Our apparatus reproduces some of the dynamic properties of the F_1_–ATP synthase motor. In the absence of ATP and at very low ATP concentrations, F_1_-ATP molecules show rotary random walks on a circle with dwells at discrete docking sites, featuring both clockwise and counterclockwise rotor movements (*19*, *20*). In contrast to the ATP synthase when supplied with ATP, the rotary Brownian motion of our prototype currently cannot be rectified. We see three possible directions for powering the motor: (i) cyclically altering the periodic energy landscape for rotor docking through, for example, laser heating according to Brownian motor concepts (*22*); (ii) using ion flow across membranes as in F_1_F_0_–ATP synthases; and (iii) coupling an out-of-equilibrium chemical reaction to rotor docking and undocking to drive the rotor. Realizing any of these possibilities will require design modifications and the development of appropriate experimental context.

Because of its modular construction and the ease of modifying any of its components, our apparatus may serve as an experimental platform toward implementing and testing various physical/chemical concepts for rectifying Brownian motion. Beyond creating rotary motors, again because of the modularity of the apparatus, it may also enable new types of single-molecule assays for receptor-ligand binding kinetics, for example, by installing receptors in the bearing cavity and their ligands on the rotor. We also observed that our assembly process proved sufficiently robust to enable the fabrication of five distinct variants of our rotary apparatus. Hence, our methods will also support creating other complex nanoscale mechanisms based on tightly fitting, sterically constrained, but mobile, DNA components.

## MATERIALS AND METHODS

### Design of scaffolded DNA origami objects

Objects were designed in an iterative procedure using caDNAno v0.2 (*16*) and CanDo (*23*, *24*).

### Molecular self-assembly with scaffolded DNA origami and polymerization protocol

All reaction mixtures contained single-stranded scaffold DNA [prepared as previously described by Kick *et al.* (*25*)] at a concentration of 50 nM and oligonucleotide strands (synthesized by Eurofins Genomics; HPSF purification) at 200 nM each. The reaction buffer includes 5 mM tris, 1 mM EDTA, 5 mM NaCl (pH 8), and 20 mM MgCl_2_. All reaction mixtures were subjected to a thermal annealing ramp using Tetrad (MJ Research, now Bio-Rad) thermal cycling devices.

During annealing, the reaction mixture was exposed to 65°C for 15 min and then the temperature was decreased by 1°C every 2 hours down to 40°C. The entire process of thermal annealing required an overall time of 22 hours 15 min. Excess oligonucleotides were subsequently removed via PEG precipitation (*26*).

The protocol used for polymerization of the rotary apparatus is as follows: (i) incubation of the rotor with the first clamp element in a stoichiometry of 1:1 in the presence of 30 mM MgCl_2_ at 20°C for 24 hours, (ii) addition of the second clamp element in a stoichiometry of 1:2 and increasing the MgCl_2_ concentration to 40 mM and incubation at 40°C for 24 hours, and (iii) addition of auxiliary oligonucleotides for fixing the brackets in a stoichiometry of 5:1 excess per strand and binding site and incubation at 20°C for 24 hours.

### Gel electrophoresis and image processing

Folded and polymerized DNA nanostructures were electrophoresed on 1.5 or 2% agarose gels containing 0.5× tris-borate EDTA and MgCl_2_ at different concentrations for 2 to 3 hours at a 70-V bias voltage in a gel box immersed in an iced water bath. The electrophoresed agarose gels were stained with ethidium bromide and scanned using a Typhoon FLA 9500 laser scanner (GE Healthcare) at a resolution of 50 μm/pixel.

### TEM and image processing

Purified reaction products were adsorbed on glow-discharged formvar-supported carbon-coated Cu400 TEM grids (Science Services) and stained using a 2% aqueous uranyl formate solution containing 25 mM sodium hydroxide. Imaging was performed using a Philips CM100 electron microscope operated at 100 kV. Images were acquired using an AMT 4-megapixel charge-coupled device (CCD) camera. Micrograph scale bars were calibrated by imaging two-dimensional catalase crystals and using the lattice constants as a length reference. Imaging was performed at 28,500-fold magnification. For image processing, libraries of individual particle micrographs were created by particle picking using the EMAN2 (*27*) boxing routine. Generation of average particle micrographs was performed using the Xmipp mlf_2Dalign routine (*28*, *29*).

### TIRF microscope preparation of PEGylated microscope cover slides

The cover slides were cleaned first in 2 M NaOH for about 30 min and second by sequential sonication in 2% Hellmanex and ethanol (99%). All chemical treatment steps were followed by rinsing and sonicating in double-distilled water (ddH_2_O). Slides were flushed with N_2_ and dried for 1 hour at 70°C. A solution of 0.56% mPEG-silane and 0.005% bioPEG-silane (solved in ethanol) was then mixed in a 1:1000 ratio. Acetic acid was added to a final concentration of 1%. The previously cleaned slides were incubated with the prepared solution for 30 min at 70°C. They were then cleaned with ddH_2_O and flushed with N_2_ for drying. The slides are stored protected from light to avoid light-induced degradation.

### Sample preparation and single-molecule microscopy experiments

All samples used for single-molecule microscopy experiments were purified from agarose gels after polymerization. To create a small-sample chamber, a glass coverslip was attached to the PEGylated cover slider by vacuum grease. This chamber was washed with a T50 buffer (10 mM tris, 50 mM NaCl), incubated with a solution of NeutrAvidin (0.05 mg/ml) for 10 min, and subsequently washed with the T50 buffer solution. For immobilization purposes, the sample was incubated in the chamber for 10 min and was subsequently washed with single-molecule imaging buffer [100 mM tris-HCl, 2 mM Trolox, 0.8% d-glucose, catalase (2000 U/ml), glucose oxydase (165 U/ml), 1 mM EDTA, 5 mM NaCl, and different MgCl_2_ concentration between 5 and 20 mM; all chemicals were purchased from Sigma-Aldrich].

Experiments shown in Figs. 3 and 4 were performed at ~20°C using a home-built objective-type total internal reflection single-molecule setup. The samples were excited with a red diode laser (640 nm; Oxxius). Fluorescence signals of the cyanine-5 dyes were collected through a 100× numerical aperture, 1.49 oil-immersion objective (Nikon Apochromat) and recorded by an electron-multiplying CCD (EMCCD) camera (Andor iXon+). Videos were taken with a frame rate of 50 ms.

Experiments shown in Fig. 4 were performed using the same setup in ALEX mode (*30*). An acousto-optical tunable filter (Pegasus Optics) was used to alternatingly excite the sample with a green diode laser (532 nm; Oxxius) and with a red diode laser (640 nm; Oxxius). Fluorescence signals of the cyanine-5 and cyanine-3 dyes were collected and split by wavelength with a dichroic mirror into two detection channels. The two detection channels were recorded by two separate EMCCD cameras (Andor iXon+). Videos were taken with a frame rate of 50 ms, which results in an effective time of 100 ms per frame due to ALEX. The videos were processed and analyzed using custom-made software written in MathWorks MATLAB (R2013b; 8.2.0.701).

## Supplementary Material

http://advances.sciencemag.org/cgi/content/full/2/2/e1501209/DC1

## SUPPLEMENTARY MATERIALS

Supplementary material for this article is available at http://advances.sciencemag.org/cgi/content/full/2/2/e1501209/DC1 Fig. S1. Scaffold/staple layout of the rotor unit, generated with caDNAno v0.2. Fig. S2. Scaffold/staple layout of the clamp element with one socket for rotor docking, generated with caDNAno v0.2. Fig. S3. EMA of the assembly of the rotary apparatus with 22 mM MgCl_2_. Fig. S4. Average TEM images of bearing dimer before (A) and after (B) addition of auxiliary oligonucleotides for closure of brackets. Fig. S5. EMA of the crank lever extension. Fig. S6. Exemplary TEM micrographs of the extended crank lever version of the rotary apparatus. Fig. S7. Comparison of evanescent (A) and epi-illumination excitation (B) measurements acquired in the presence of 5 mM MgCl_2_. Fig. S8. Scaffold/staple layout of the six-helix bundle used as a crank lever extension, generated with caDNAno v0.2. Fig. S9. All 1500 consecutive frames of the single-particle video discussed in Fig. 3D, shown in the order of acquisition from left to right, top to bottom. Fig. S10. All 1500 consecutive frames of the single-particle video discussed in Fig. 3E, shown in the order of acquisition from left to right, top to bottom. Fig. S11. EMA of the assembly of the static variant with 11 mM MgCl_2_ (A) and the weak variant with 22 mM MgCl_2_ (B) of the rotary apparatus. Fig. S12. All 750 frames of the single-particle recording discussed in Fig. 4A, shown in the order of acquisition from left to right, top to bottom. Fig. S13. Exemplary switching single-particle recordings of short crank lever version calculated as in Fig. 4 (B and C). Fig. S14. Scaffold/staple layout of the static version, generated with caDNAno v0.2. Fig. S15. Scaffold/staple layout of the weak rotor unit, generated with caDNAno v0.2. Fig. S16. Scaffold/staple layout of the clamp element with three docking positions, generated with caDNAno v0.2. Fig. S17. EMA of the assembly of the rotary apparatus with six docking positions. Movie S1. Schematic animation of the polymerization steps of the rotary apparatus without crank lever extension. Movie S2. Schematic animation of the freely rotating rotary apparatus without crank lever extension. Movie S3. Schematic animation of the freely rotating rotary apparatus with crank lever extension. Movie S4. Single-particle fluorescence microscopy recording discussed in Fig. 3D. Movie S5. Single-particle fluorescence microscopy recording discussed in Fig. 3E. Movie S6. Single-particle fluorescence microscopy recording discussed in Fig. 4A.
