# Supplementary material for: Nanoscale rotary apparatus formed from tight-fitting 3D DNA components
Source: Sci Adv. 2016 Feb 19;2(2):e1501209. doi: 10.1126/sciadv.1501209 (PMC4788491; doi:10.1126/sciadv.1501209)
Supplement: http://advances.sciencemag.org/cgi/content/full/2/2/e1501209/DC1 [file supp_2_2_e1501209__index.html]

Science Advances | Science Advances

## Supplementary Materials

**This PDF file includes:**

- Fig. S1. Scaffold/staple layout of the rotor unit, generated with caDNAno v0.2.
- Fig. S2. Scaffold/staple layout of the clamp element with one socket for rotor  
   docking, generated with caDNAno v0.2.
- Fig. S3. EMA of the assembly of the rotary apparatus with 22 mM MgCl2.
- Fig. S4. Average TEM images of bearing dimer before (A) and after (B) addition  
   of auxiliary oligonucleotides for closure of brackets.
- Fig. S5. EMA of the crank lever extension.
- Fig. S6. Exemplary TEM micrographs of the extended crank lever version of the  
   rotary apparatus.
- Fig. S7. Comparison of evanescent (A) and epi-illumination excitation (B)  
   measurements acquired in the presence of 5 mM MgCl2.
- Fig. S8. Scaffold/staple layout of the six-helix bundle used as a crank lever  
   extension, generated with caDNAno v0.2.
- Fig. S9. All 1500 consecutive frames of the single-particle video discussed in Fig.  
   3D, shown in the order of acquisition from left to right, top to bottom.
- Fig. S10. All 1500 consecutive frames of the single-particle video discussed in  
   Fig. 3E, shown in the order of acquisition from left to right, top to bottom.
- Fig. S11. EMA of the assembly of the static variant with 11 mM MgCl2 (A) and  
   the weak variant with 22 mM MgCl2 (B) of the rotary apparatus.
- Fig. S12. All 750 frames of the single-particle recording discussed in Fig. 4A,  
   shown in the order of acquisition from left to right, top to bottom.
- Fig. S13. Exemplary switching single-particle recordings of short crank lever  
   version calculated as in Fig. 4 (B and C).
- Fig. S14. Scaffold/staple layout of the static version, generated with caDNAno  
   v0.2.
- Fig. S15. Scaffold/staple layout of the weak rotor unit, generated with caDNAno  
   v0.2.
- Fig. S16. Scaffold/staple layout of the clamp element with three docking  
   positions, generated with caDNAno v0.2.
- Fig. S17. EMA of the assembly of the rotary apparatus with six docking positions.

Download PDF

**Other Supplementary Material for this manuscript includes the following:**

- Movie S1 (.mov format). Schematic animation of the polymerisation steps of the  
   rotary apparatus without crank lever extension.
- Movie S2 (.mov format). Schematic animation of the freely rotating rotary  
   apparatus without crank lever extension.
- Movie S3 (.mov format). Schematic animation of the freely rotating rotary  
   apparatus with crank lever extension.
- Movie S4 (.avi format). Single-particle fluorescence microscopy recording  
   discussed in Fig. 3D.
- Movie S5 (.avi format). Single-particle fluorescence microscopy recording  
   discussed in Fig. 3E.
- Movie S6 (.avi format). Single-particle fluorescence microscopy recording  
   discussed in Fig. 4A.

**Files in this Data Supplement:**

- Adobe PDF - 1501209\_SM.pdf
