# Supplementary material for: Nanoscale rotary apparatus formed from tight-fitting 3D DNA components
Source: Sci Adv. 2016 Feb 19;2(2):e1501209. doi: 10.1126/sciadv.1501209 (PMC4788491; doi:10.1126/sciadv.1501209)
Supplement: http://advances.sciencemag.org/cgi/content/full/2/2/e1501209/DC1 [file 1501209_SM.pdf]

Science Advances

AAAS

advances.sciencemag.org/cgi/content/full/2/2/e1501209/DC1

Supplementary Materials for

Nanoscale rotary apparatus formed from tight-fitting 3D DNA components

Philip Ketterer, Elena M. Willner, Hendrik Dietz

Published 19 February 2016, *Sci. Adv.* **2**, e1501209 (2016)
DOI: 10.1126/sciadv.1501209

This PDF file includes:

Fig. S1. Scaffold/staple layout of the rotor unit, generated with caDNAno v0.2.
Fig. S2. Scaffold/staple layout of the clamp element with one socket for rotor docking, generated with caDNAno v0.2.
Fig. S3. EMA of the assembly of the rotary apparatus with 22 mM $MgCl_2$.
Fig. S4. Average TEM images of bearing dimer before (A) and after (B) addition of auxiliary oligonucleotides for closure of brackets.
Fig. S5. EMA of the crank lever extension.
Fig. S6. Exemplary TEM micrographs of the extended crank lever version of the rotary apparatus.
Fig. S7. Comparison of evanescent (A) and epi-illumination excitation (B) measurements acquired in the presence of 5 mM $MgCl_2$.
Fig. S8. Scaffold/staple layout of the six-helix bundle used as a crank lever extension, generated with caDNAno v0.2.
Fig. S9. All 1500 consecutive frames of the single-particle video discussed in Fig. 3D, shown in the order of acquisition from left to right, top to bottom.
Fig. S10. All 1500 consecutive frames of the single-particle video discussed in Fig. 3E, shown in the order of acquisition from left to right, top to bottom.
Fig. S11. EMA of the assembly of the static variant with 11 mM $MgCl_2$ (A) and the weak variant with 22 mM $MgCl_2$ (B) of the rotary apparatus.
Fig. S12. All 750 frames of the single-particle recording discussed in Fig. 4A, shown in the order of acquisition from left to right, top to bottom.
Fig. S13. Exemplary switching single-particle recordings of short crank lever version calculated as in Fig. 4 (B and C).
Fig. S14. Scaffold/staple layout of the static version, generated with caDNAno v0.2.

Fig. S15. Scaffold/staple layout of the weak rotor unit, generated with caDNAno v0.2.
Fig. S16. Scaffold/staple layout of the clamp element with three docking positions, generated with caDNAno v0.2.
Fig. S17. EMA of the assembly of the rotary apparatus with six docking positions.

Other Supplementary Material for this manuscript includes the following:
(available at advances.sciencemag.org/cgi/content/full/2/2/e1501209/DC1)

Movie S1 (.mov format). Schematic animation of the polymerisation steps of the rotary apparatus without crank lever extension.
Movie S2 (.mov format). Schematic animation of the freely rotating rotary apparatus without crank lever extension.
Movie S3 (.mov format). Schematic animation of the freely rotating rotary apparatus with crank lever extension.
Movie S4 (.avi format). Single-particle fluorescence microscopy recording discussed in Fig. 3D.
Movie S5 (.avi format). Single-particle fluorescence microscopy recording discussed in Fig. 3E.
Movie S6 (.avi format). Single-particle fluorescence microscopy recording discussed in Fig. 4A.

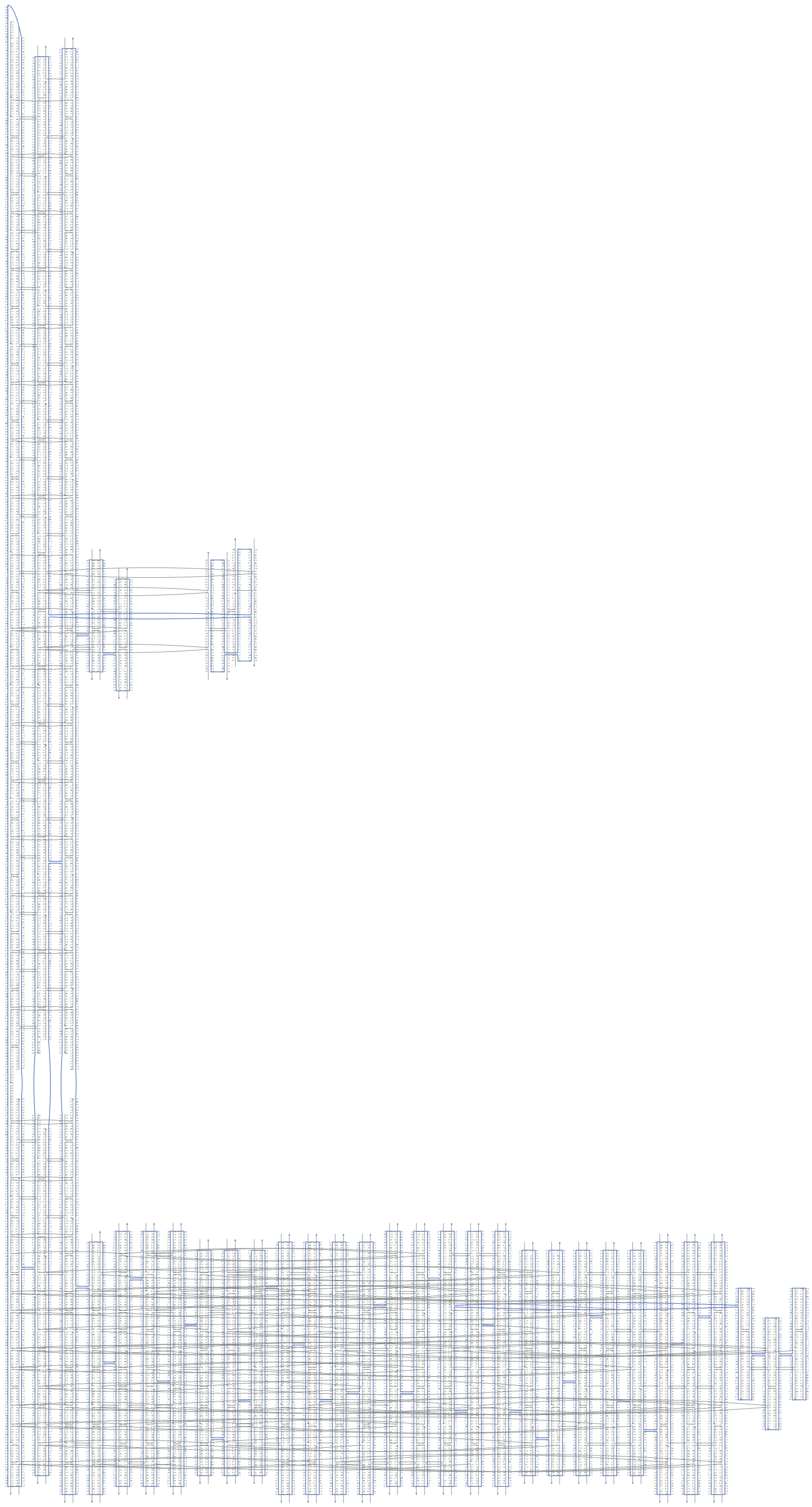

Fig S1. Scaffold/staple layout of the rotor unit, generated with caDNAno v0.2.

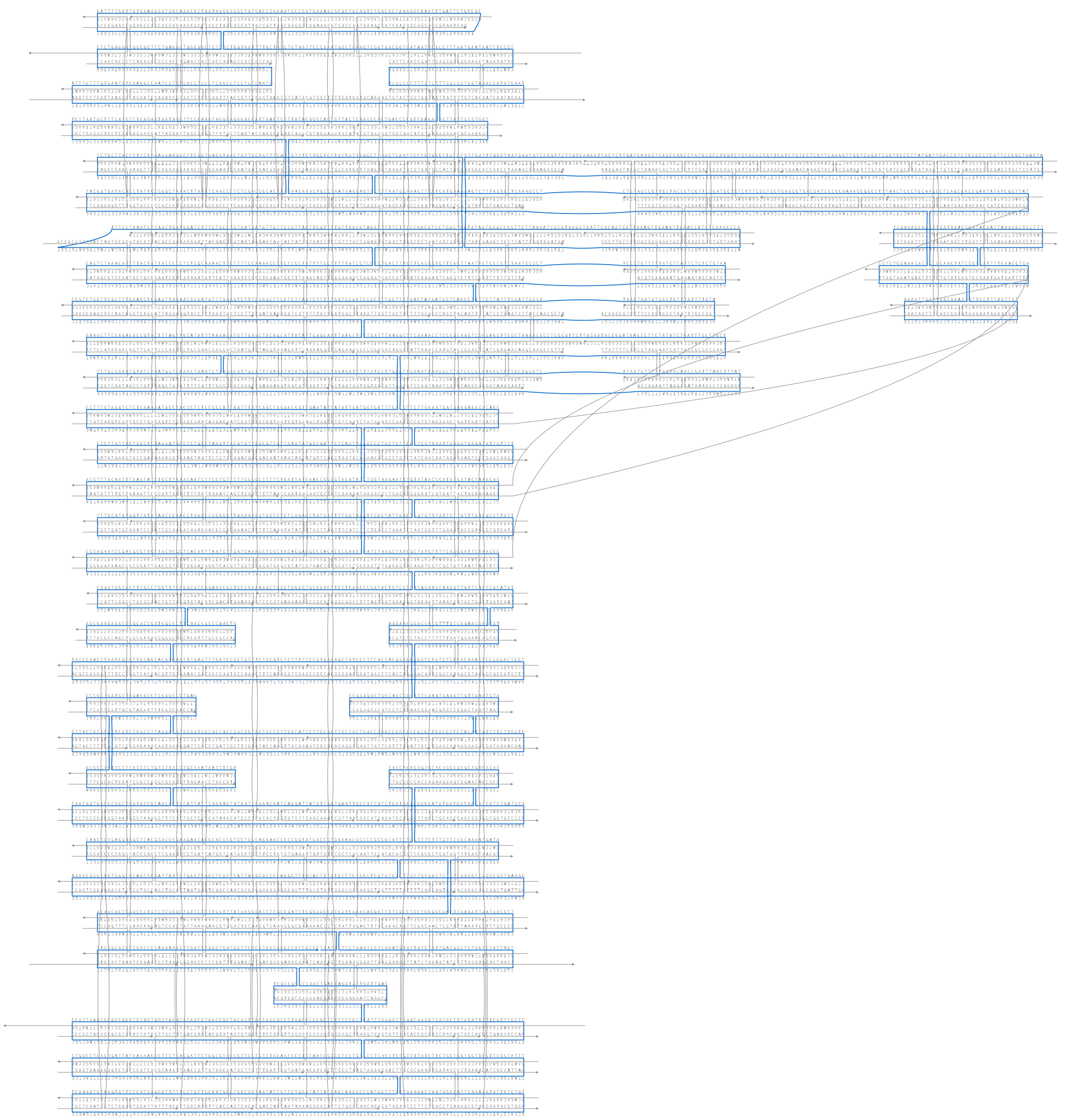

Fig. S2. Scaffold/staple layout of the clamp element with one socket for rotor docking, generated with caDNAno v0.2.

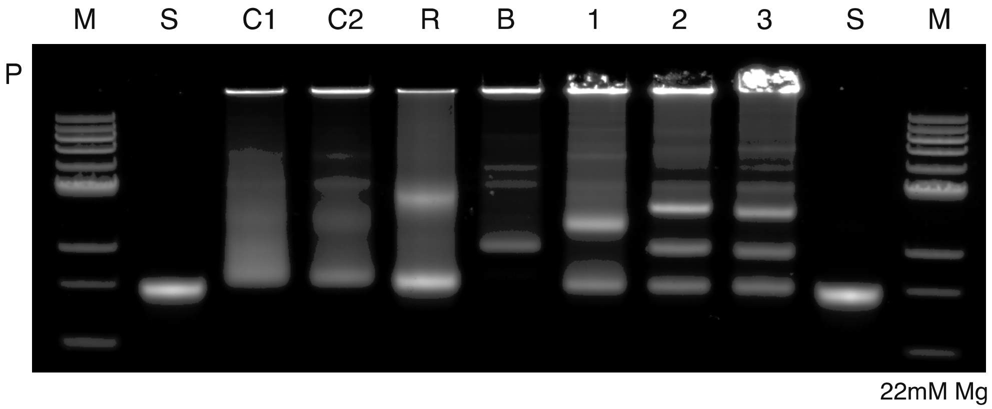

Fig. S3. **EMA of the assembly of the rotary apparatus with 22 mM $MgCl_2$.** Laser-scanned image of a 2% agarose run on an iced water-bath on which the following samples were electrophoresed: M = 1kb ladder, S = scaffold DNA, C1 = clamp element 1, C2 = clamp element 2, R = rotor unit, B = bearing dimer, 1 = rotor and clamp dimerization, 2 = bearing closure reaction in which the second clamp element was added to the mixture in 1, 3 = bracket closure reaction in which auxiliary oligonucleotides were added to the mixture in 2. P denotes the pocket.

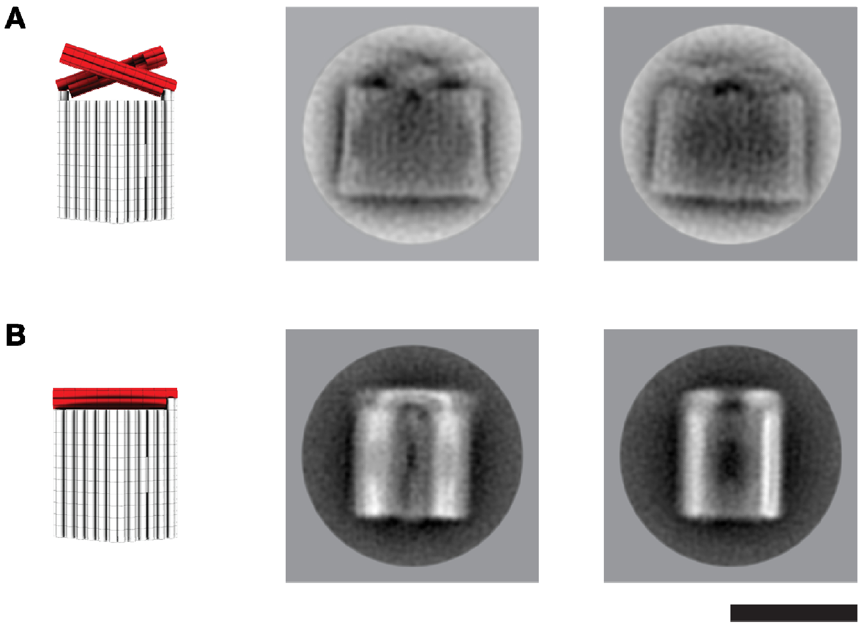

Fig. S4. Average TEM images of bearing dimer before (**A**) and after (**B**) addition of auxiliary oligonucleotides for closure of brackets. Scale bar is 50 nm.

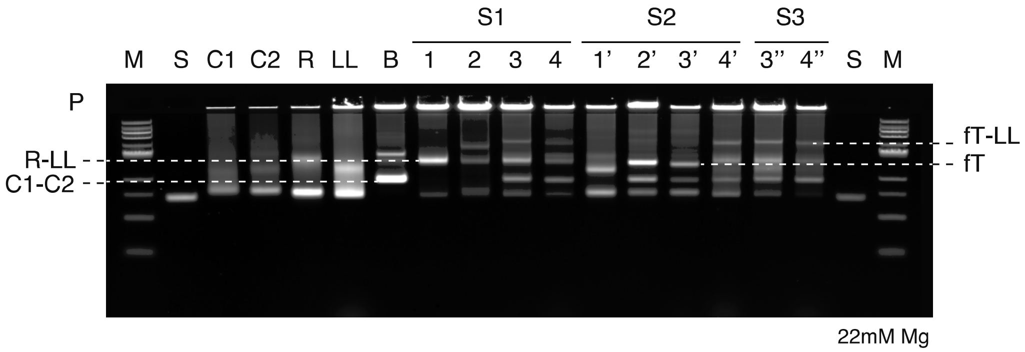

Fig. S5. EMA of the crank lever extension. Laser-scanned image of a 1.5% agarose gels with 22 mM $MgCl_2$ run on an iced water-bath. M = 1kb ladder, S = scaffold DNA, C1 = clamp element 1, C2 = clamp element 2, R = rotor unit, LL = lever extension, B = bearing dimer, S1 = assembly strategy 1, 1 = rotor and lever extension dimerization, 2 = rotor and clamp dimerization, 3 = bearing closure reaction in which the second clamp element was added to the mixture in 2, 4 = bracket closure reaction in which auxiliary oligonucleotides were added to the mixture in 3, S2 = assembly strategy 2, 1’–3’ = trimerization as in Supplementary Fig. 3, 4’ = tetramerization with the lever extension in which the lever extension was added to the mixture in 3’, S3 = assembly strategy 3, 3’’ = tetramerization with the lever extension in which the lever extension was added to the mixture in 2’, 4’’ = bracket closure reaction in which auxiliary oligonucleotides were added to the mixture in 3’’. P denotes the pocket, C1-C2 the dimer consisting of both clamp elements, R-LL the dimer consisting of rotor unit and lever extension, fT the trimer consisting of rotor unit and both clamp elements with fixed brackets, and fT-LL the tetramer consisting of fT and the lever extension. S3 was used for experimental data shown in Fig. 3.

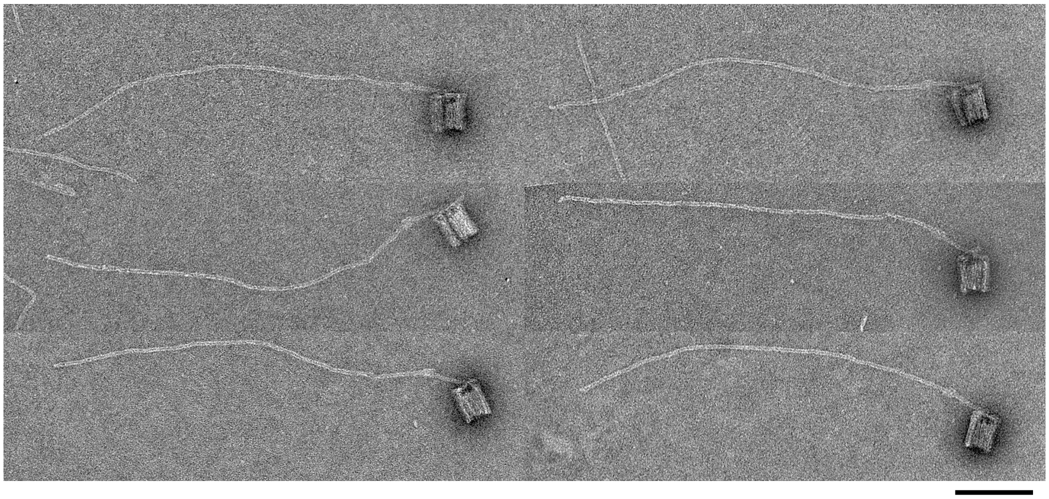

Fig. S6. Exemplary TEM micrographs of the extended crank lever version of the rotary apparatus. Scale bar is 100 nm.

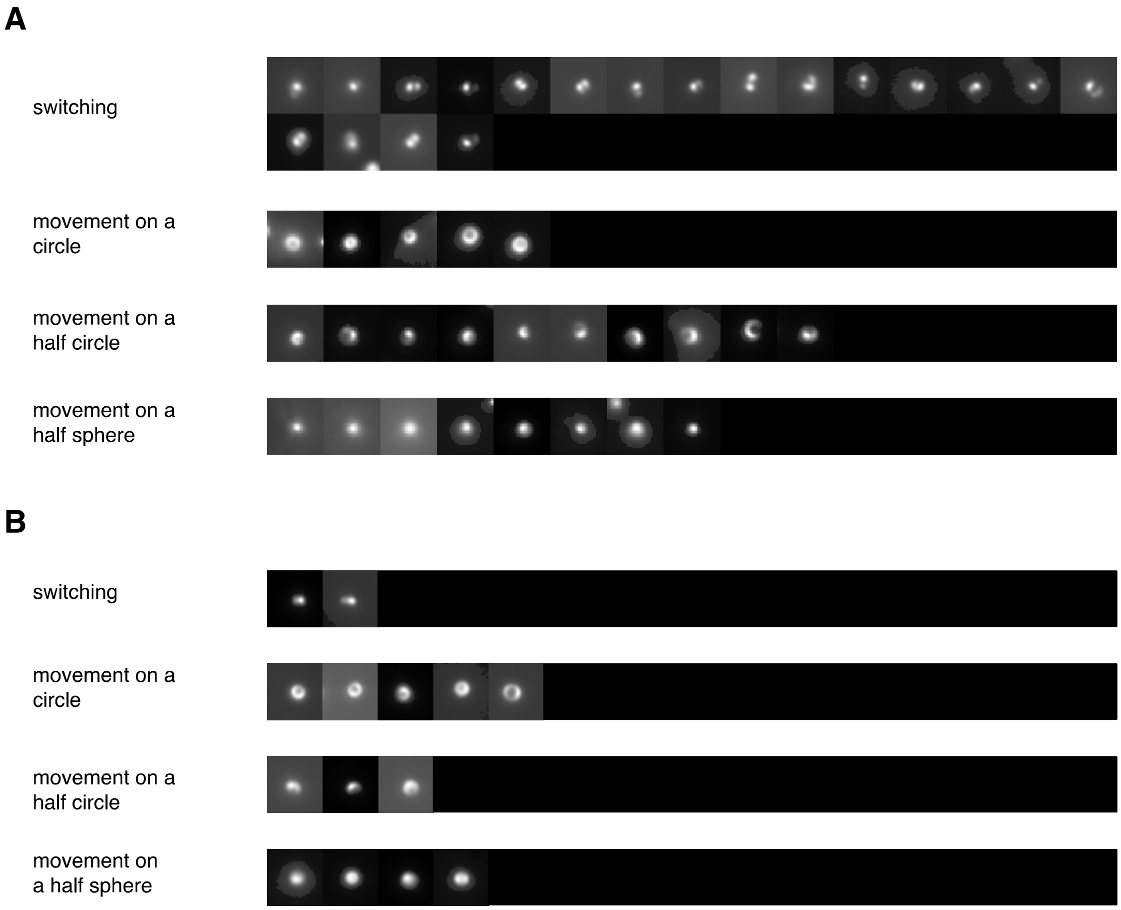

Fig. S7. Comparison of evanescent (A) and epi-illumination excitation (B) measurements acquired in the presence of 5 mM $MgCl_2$. Shown are sums over all 1500 frames per single particle video. Exposure time is 50 ms per frame.

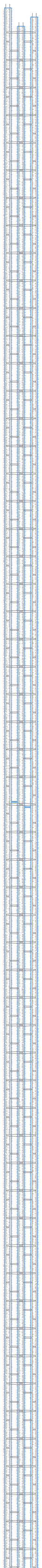

Fig. S8. Scaffold/staple layout of the six-helix bundle used as a crank lever extension, generated with caDNAno v0.2.

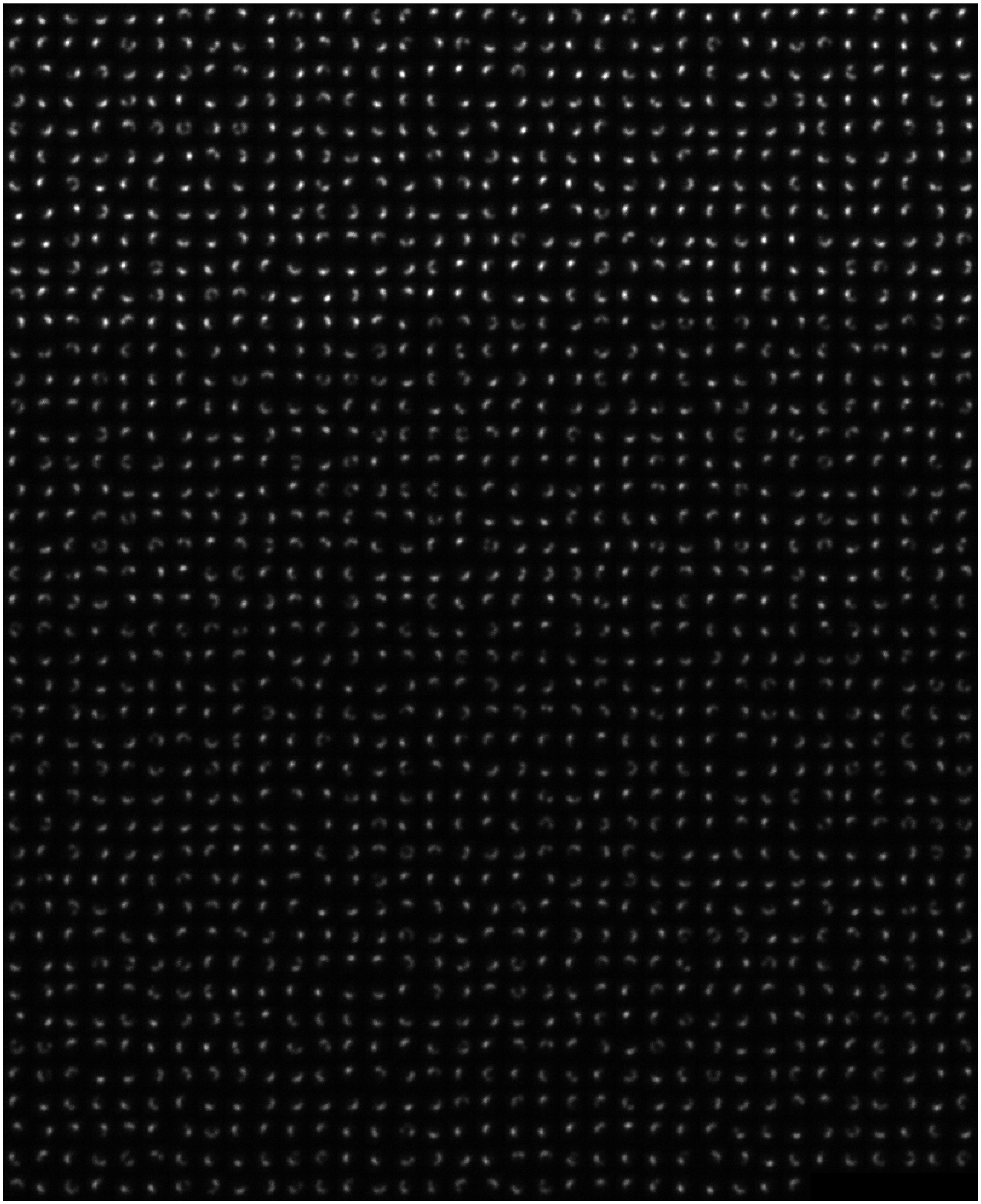

Fig. S9. All 1500 consecutive frames of the single-particle video discussed in Fig. 3D, shown in the order of acquisition left to right, top to bottom.

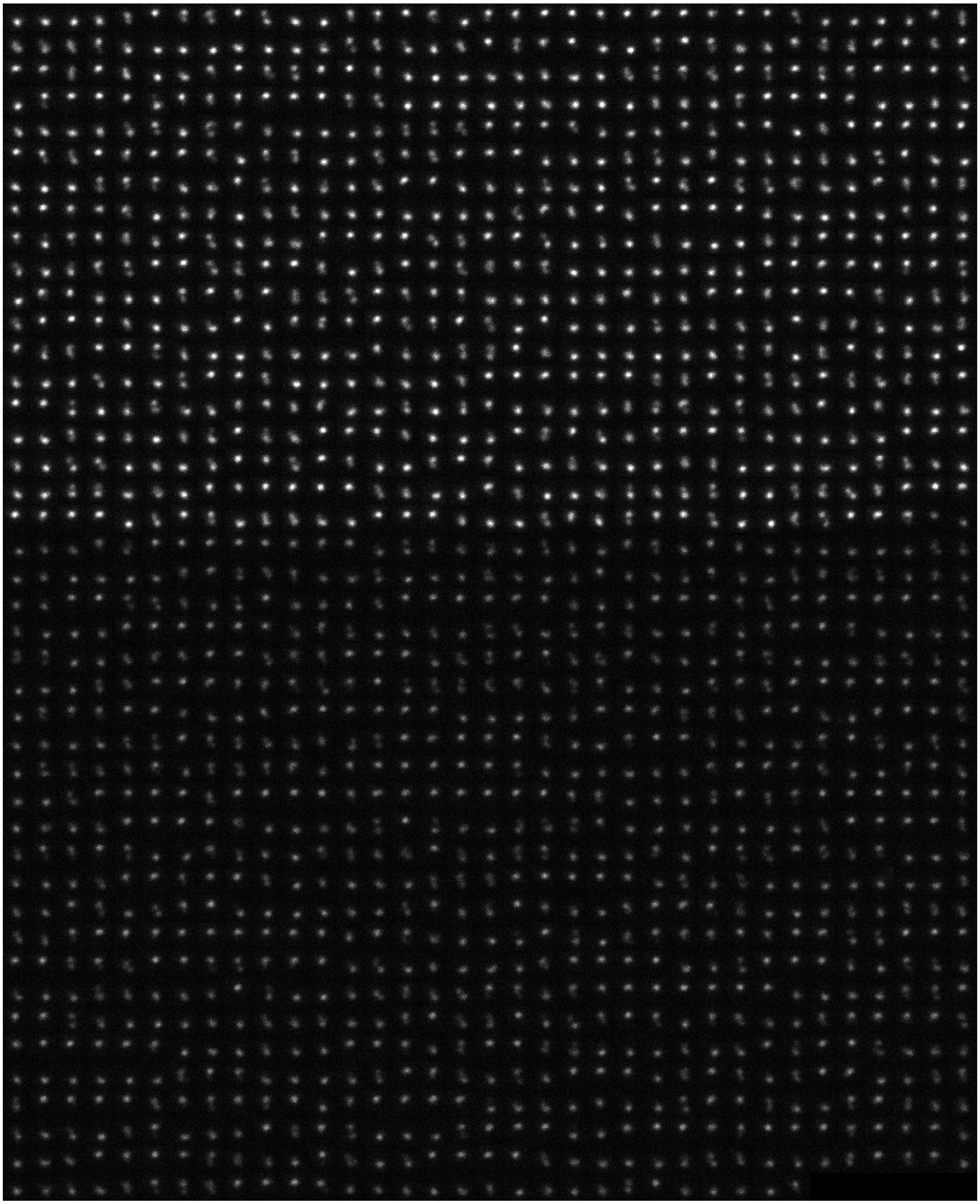

Fig. S10. All 1500 consecutive frames of the single-particle video discussed in Fig. 3E, shown in the order of acquisition left to right, top to bottom.

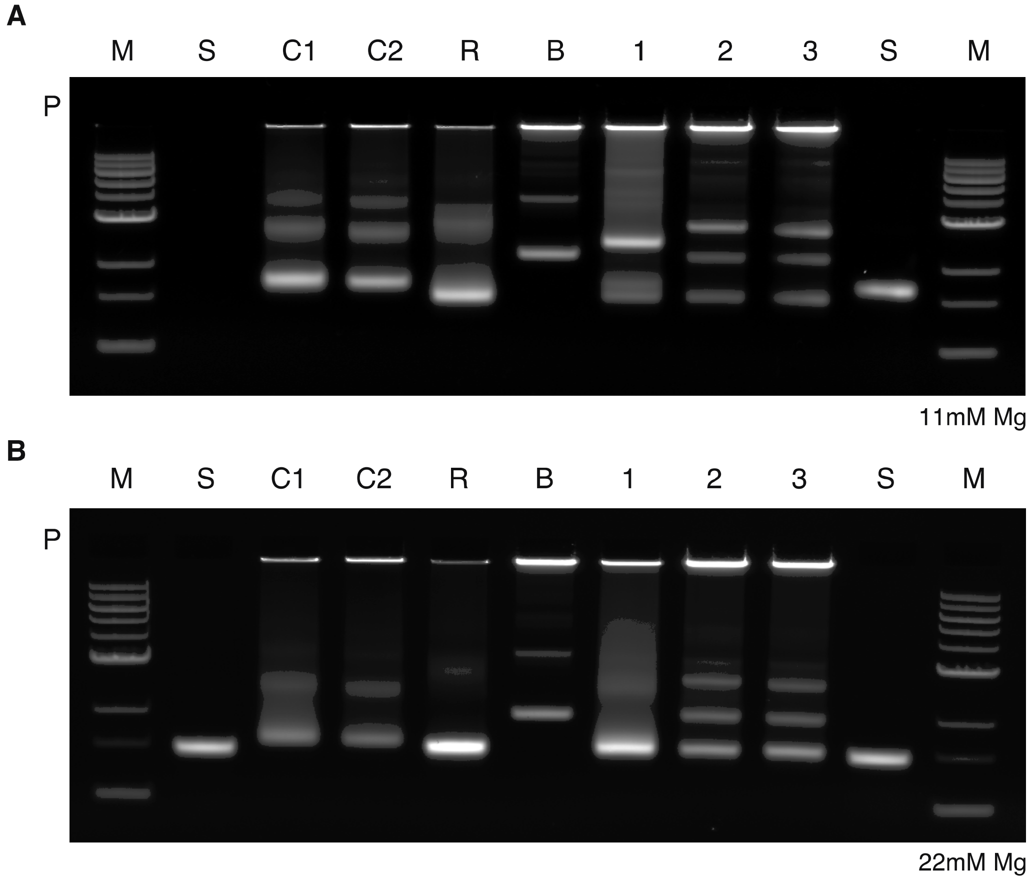

Fig. S11. EMA of the assembly of the static variant with 11 mM $MgCl_2$ (A) and the weak variant with 22 mM $MgCl_2$ (B) of the rotary apparatus. Laser-scanned images of 2% agarose run on an iced water-bath on which the following samples were electrophoresed: M = 1kb ladder, S = scaffold DNA, C1 = clamp element 1, C2 = clamp element 2, R = rotor unit, B = bearing dimer, 1 = rotor and clamp dimerization, 2 = bearing closure reaction in which the second clamp element was added to the mixture in 1, 3 = bracket closure reaction in which auxiliary oligonucleotides were added to the mixture in 2. P denotes the pocket.

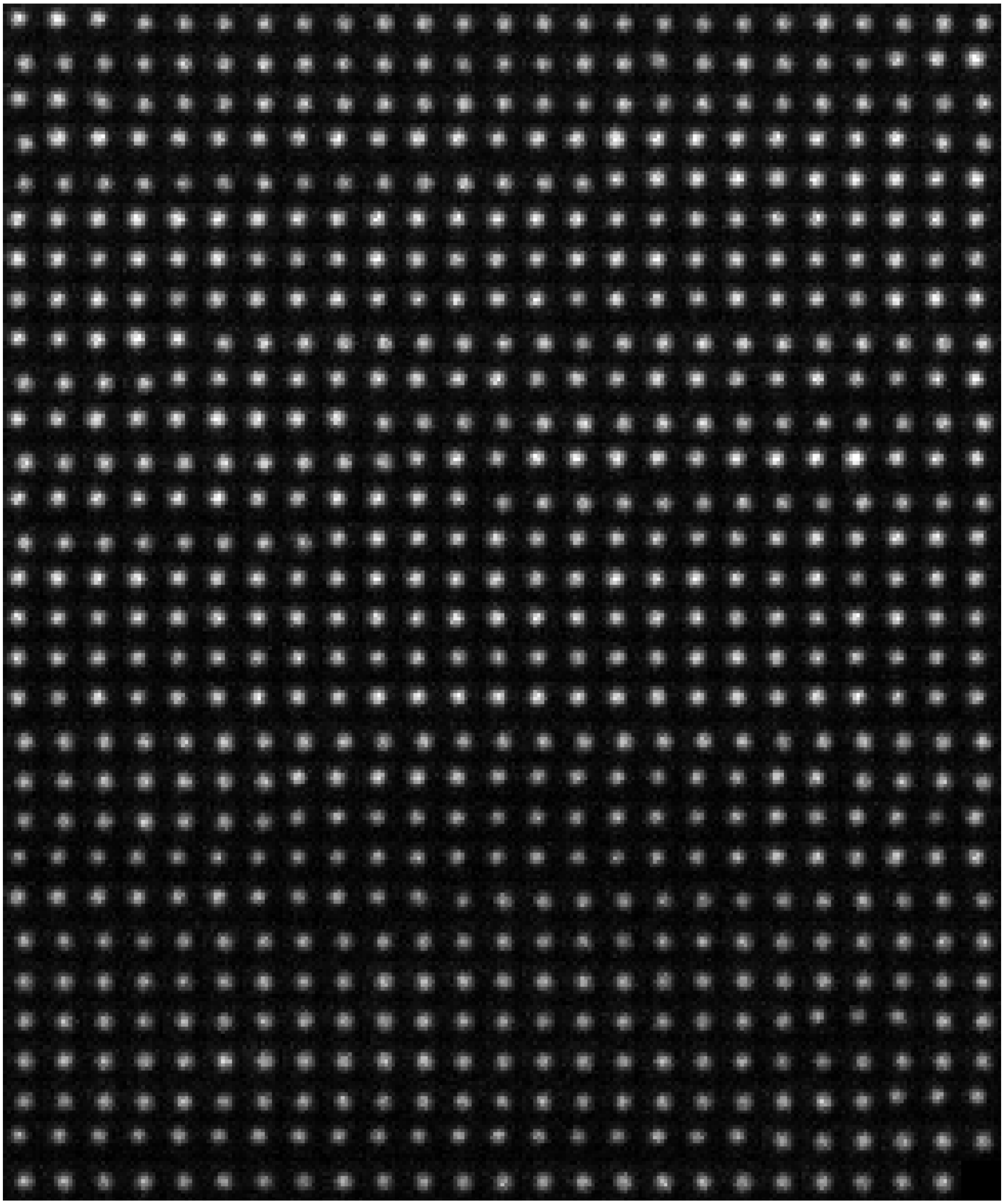

Fig. S12. All 750 frames of the single-particle recording discussed in Fig. 4A, shown in the order of acquisition left to right, top to bottom.

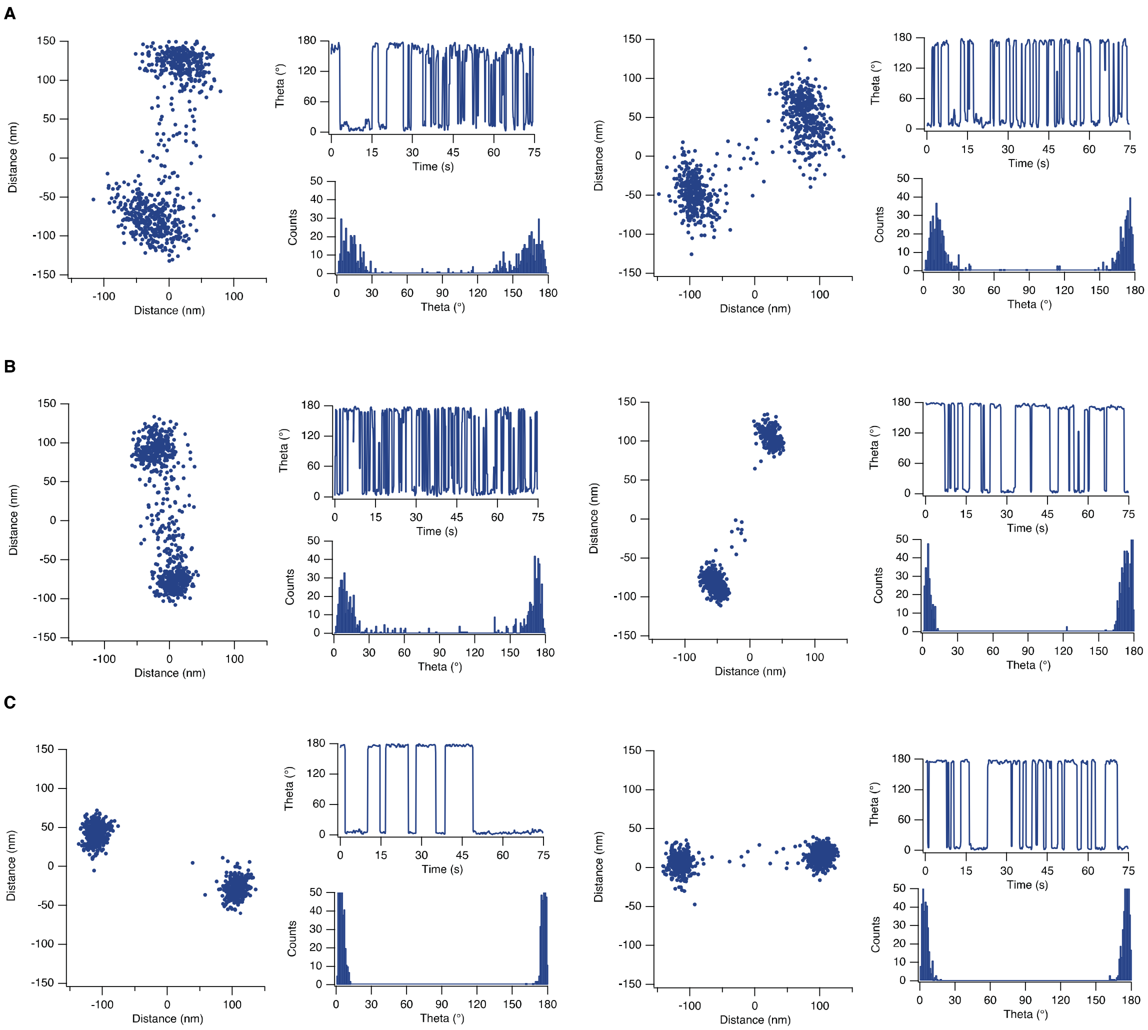

Fig. S13. Exemplary switching single-particle recordings of short crank lever version calculated as in Fig. 4 (B and C). (**A**), strong version in the presence of 5 mM $MgCl_2$. (**B**), weak version in the presence of 5 mM $MgCl_2$. (**C**), weak version in the presence of 10 mM $MgCl_2$.

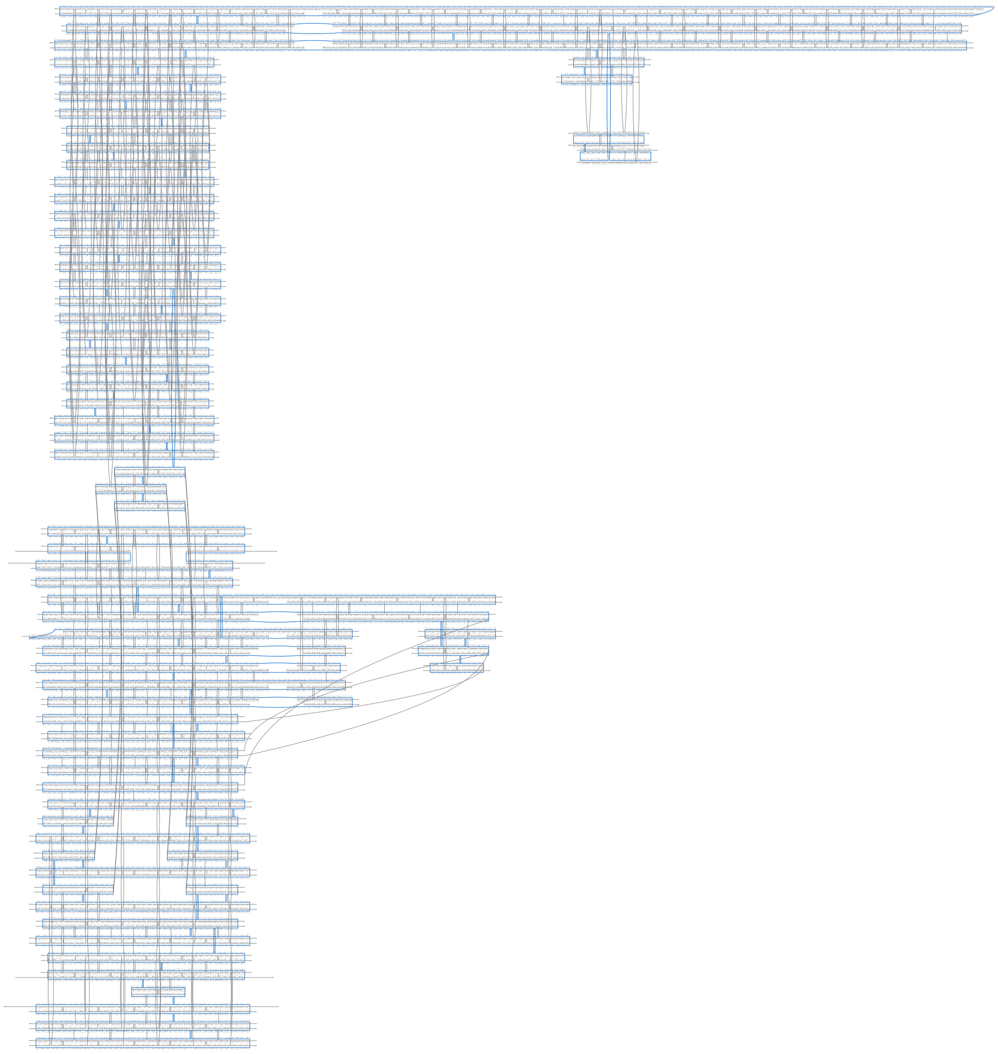

Fig. S14. Scaffold/staple layout of the static version, generated with caDNAno v0.2.

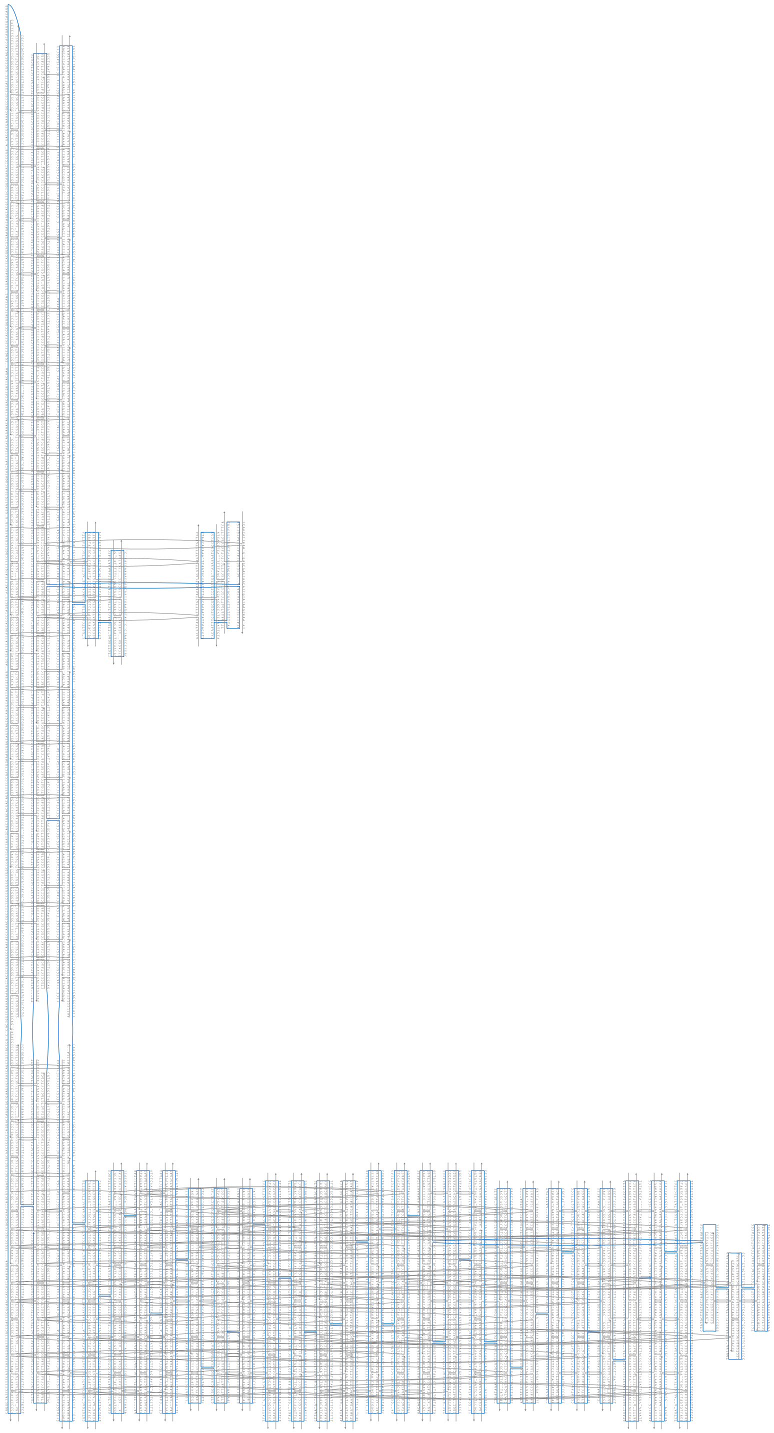

Fig. S15. Scaffold/staple layout of the weak rotor unit, generated with caDNAno v0.2.

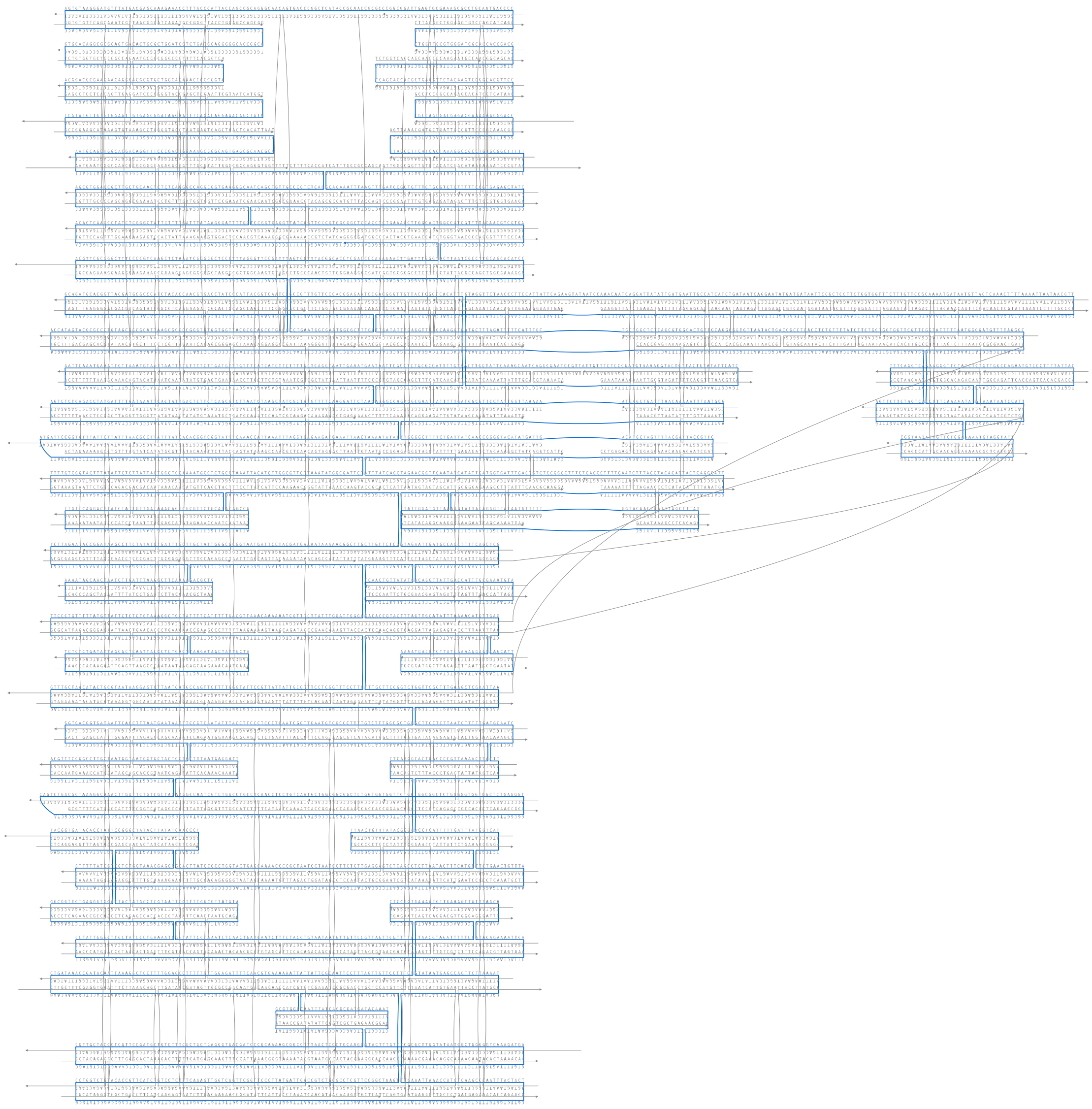

Fig. S16. Scaffold/staple layout of the clamp element with three docking positions, generated with caDNAno v0.2.

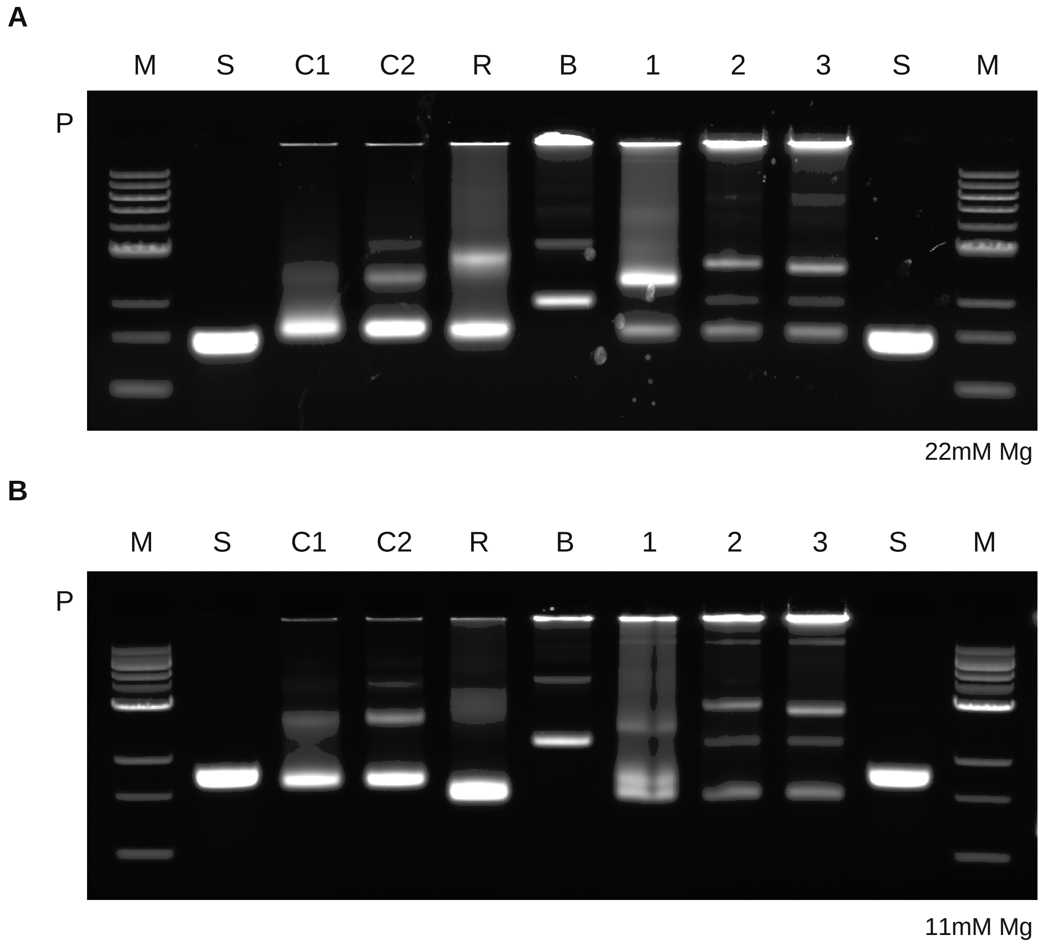

Fig. S17. EMA of the assembly of the rotary apparatus with six docking positions. Laser-scanned images of 2% agarose gels with 22 mM (**A**) and 11 mM $MgCl_2$ (**B**) run on an iced water-bath on which the following samples were electrophoresed: M = 1kb ladder, S = scaffold DNA, C1 = clamp element 1, C2 = clamp element 2, R = rotor unit, B = bearing dimer, 1 = rotor and clamp dimerization, 2 = bearing closure reaction in which the second clamp element was added to the mixture in 1, 3 = bracket closure reaction in which auxiliary oligonucleotides were added to the mixture in 2. P denotes the pocket.
